# 

**Published:** 1905-09

**Authors:** 


					﻿Sixty-first Year.
Vol. LXI.
SEPTEMBER, 1905.
No. 2
BUFFALO
MEDICAL JQWfcU
Established 1845 by AUSTIlfe. FLINT.. D.
EDITOR :
WILLIAM WARREN POTTER, M. D.
ASSISTANT EDITORS:
WILLIAM C. KRAUSS, M. D.
NELSON W. WILSON, M. D.
ASSOCIATE EDITORS:
JAMES WRIGHT PUTNAM, M. D.
JOHN PARMENTER, M. D.
HARVEY R. GAYLORD, M. D.
ERNEST WENDE, M. D.
JOHN A. MILLER, Ph. D.
MAUD J. FRYE, M. p.
Fn<* terms and other information relating to the Journal, seepage ▼!.
				

